# Global Importance of Hydroxymethanesulfonate in Ambient Particulate Matter: Implications for Air Quality

**DOI:** 10.1029/2020JD032706

**Published:** 2020-09-11

**Authors:** Jonathan M. Moch, Eleni Dovrou, Loretta J. Mickley, Frank N. Keutsch, Zirui Liu, Yuesi Wang, Tracy L. Dombek, Mikinori Kuwata, Sri Hapsari Budisulistiorini, Liudongqing Yang, Stefano Decesari, Marco Paglione, Becky Alexander, Jingyuan Shao, J. William Munger, Daniel J. Jacob

**Affiliations:** ^1^ Department of Earth and Planetary Sciences Harvard University Cambridge MA USA; ^2^ John A. Paulson School of Engineering and Applied Sciences Harvard University Cambridge MA USA; ^3^ Department of Chemistry and Chemical Biology Harvard University Cambridge MA USA; ^4^ State Key Laboratory of Atmospheric Boundary Layer Physics and Atmospheric Chemistry, Institute of Atmospheric Physics Chinese Academy of Sciences Beijing China; ^5^ Analytical Sciences Division, RTI International, Research Triangle Park Durham NC USA; ^6^ Asian School of the Environment and Earth Observatory of Singapore Nanyang Technological University Singapore; ^7^ Now in the Department of Atmospheric and Oceanic Sciences, School of Physics, and BIC‐ESAT Peking University Beijing China; ^8^ Now in Wolfson Atmospheric Chemistry Laboratories, Department of Chemistry University of York York UK; ^9^ Italian National Research Council ‐ Institute of Atmospheric Sciences and Climate (CNR‐ISAC) Bologna Italy; ^10^ Department of Atmospheric Sciences University of Washington WA USA; ^11^ College of Flying Technology Civil Aviation University of China Tianjin China

**Keywords:** air pollution, hydroxymethanesulfonate, sulfate, formaldehyde, cloud chemistry, aerosols

## Abstract

Sulfur compounds are an important constituent of particulate matter, with impacts on climate and public health. While most sulfur observed in particulate matter has been assumed to be sulfate, laboratory experiments reveal that hydroxymethanesulfonate (HMS), an adduct formed by aqueous phase chemical reaction of dissolved HCHO and SO_2_, may be easily misinterpreted in measurements as sulfate. Here we present observational and modeling evidence for a ubiquitous global presence of HMS. We find that filter samples collected in Shijiazhuang, China, and examined with ion chromatography within 9 days show as much as 7.6 μg m^−3^ of HMS, while samples from Singapore examined 9–18 months after collection reveal ~0.6 μg m^−3^ of HMS. The Shijiazhuang samples show only minor traces of HMS 4 months later, suggesting that HMS had decomposed over time during sample storage. In contrast, the Singapore samples do not clearly show a decline in HMS concentration over 2 months of monitoring. Measurements from over 150 sites, primarily derived from the IMPROVE network across the United States, suggest the ubiquitous presence of HMS in at least trace amounts as much as 60 days after collection. The degree of possible HMS decomposition in the IMPROVE observations is unknown. Using the GEOS‐Chem chemical transport model, we estimate that HMS may account for 10% of global particulate sulfur in continental surface air and over 25% in many polluted regions. Our results suggest that reducing emissions of HCHO and other volatile organic compounds may have a co‐benefit of decreasing particulate sulfur.

## Key Points

- New and reanalyzed observations suggest a ubiquitous global presence of hydroxymethanesulfonate (HMS) in particulate matter
- GEOS‐Chem simulations suggest HMS may comprise over 25% of particulate sulfur in many polluted regions, especially in continental winter
- Reductions of formaldehyde and other volatile organic compounds may have the co‐benefit of reducing particulate sulfur

## 1 Introduction

Particulate air pollution is a major contributor to global mortality, with over four million deaths per year attributed to fine particle pollution (PM_2.5_, particulate matter with diameter less than 2.5 μm; Cohen et al., 2017). Such pollution is also a major source of uncertainty for climate change, with forcing due to aerosol‐radiation interactions ranging between −0.85 to +0.15 W m^−2^ and even greater uncertainty in forcing due to aerosol‐cloud interactions (Myhre et al., 2013). In many regions, particulate sulfur is the first or second most important contributor by mass to total PM_2.5_ (Kim et al., 2015; Liu et al., 2018; Snider et al., 2016). It is also the dominant aerosol component of climate forcing (Myhre et al., 2013). However, models can have difficulty reproducing particulate sulfur observations especially during winter, and as a result a variety of chemical mechanisms have been proposed as nonphotochemical sources of sulfate (Alexander et al., 2009; Cheng et al., 2016; Moch et al., 2018; Shah et al., 2018; Shao et al., 2019; Wang et al., 2014, 2020; Zhang et al., 2012). Additionally, simultaneous measurements of sulfate and total elemental particulate sulfur have sometimes revealed a puzzling mismatch, given that sulfate has long been assumed to be the only particulate sulfur compound of significance (He et al., 2001; Shakya & Peltier, 2013; Tolocka & Turpin, 2012).

Recent work suggests that hydroxymethanesulfonate (HOCH_2_SO_3_^−^ or HMS), an adduct formed from the aqueous phase reaction of sulfite or bisulfite with formaldehyde (HCHO), may comprise a significant portion of observed particulate sulfur in Beijing winter haze (Moch et al., 2018; Song et al., 2019). HMS chemistry has been well characterized in the laboratory, with formation occurring from the aqueous reaction of HCHO with sulfite (SO_3_^2−^) or bisulfite (HSO_3_^−^) and decomposition occurring by reaction of HMS with OH^−^ ions (Boyce & Hoffmann, 1984; Deister et al., 1986; Kok et al., 1986; Kovacs et al., 2005; Olson & Hoffmann, 1986):

(1) $$\mathrm{HCH}O_{\left(\mathrm{aq}\right)}+{\mathrm{HSO}}_{3}^{-}\to {\text{HOCH}}_{2}{\mathrm{SO}}_{3}^{-}$$

(2) $${\text{HCHO}}_{\left(\mathrm{aq}\right)}+{\mathrm{SO}}_{3}^{2-}+H_{2}O⇄{\text{HOCH}}_{2}{\mathrm{SO}}_{3}^{-}+{\mathrm{OH}}^{-}$$

HMS can also be oxidized in cloud water by aqueous OH radicals to form sulfite anion radicals with subsequent fast conversion to sulfate (Jacob, 1986; Olson & Fessenden, 1992):

(3) HOCH2SO3−+OH·→SO·3−+HCHOaq+H2O

The study of sulfur‐aldehyde chemistry dates back to the mid‐19th century (Schiff, 1866). Dasgupta et al. (1980) reported that atmospheric SO_2_ could be quantified through stabilization of S (IV) in solution by addition of HCHO to produce HMS, followed by detection of HMS by ion chromatography. HMS was subsequently observed as a significant S (IV) species in low clouds or fog (Munger et al., 1983, 1984, 1986), and HMS chemistry was also included in some early atmospheric chemistry models (Jacob, 1986; Jacob & Hoffmann, 1983). However, despite sporadic identification of HMS in aerosols (Dixon & Aasen, 1999; Scheinhardt et al., 2014; Whiteaker & Prather, 2003), it has been mostly overlooked as a potential contributor to atmospheric sulfur since the 1980s.

Distinguishing HMS from sulfate is challenging with the most widely used measurement techniques, and this may partly account for the neglect of the HMS contribution to PM. Measurements by ion chromatography (IC) can easily misinterpret HMS as sulfate due to the difficulty in fully separating the two compounds with the most commonly used IC columns (Dovrou et al., 2019; Moch et al., 2018). In certain IC systems, HMS may also decompose and possibly oxidize to sulfate due to use of a high pH eluent (Dovrou et al., 2019). HMS may also be oxidized to sulfate during sample preparation (Ma et al., 2020). It is also difficult to quantify HMS via aerosol mass spectrometry (AMS), especially using standard approaches, since HMS fragment ions are common to many organic and sulfur species (Dovrou et al., 2019). Recent work suggests that AMS can in fact provide insight into the HMS contribution to PM if attention is paid to matrix effects and ion balance (Song et al., 2019). Other recent work has shown a high correlation between HMS identified by nuclear magnetic resonance spectroscopy (NMR) and certain oxygenated organic aerosol components identified by positive matrix factorization analysis of organic aerosol mass spectra and interpreted as SOA formed via aqueous phase processes (Gilardoni et al., 2016; Paglione et al., 2020). HMS has also been detected via electrospray ionization‐tandem and via single particle mass spectrometry, but matrix effects can impede efforts at quantification (Chapman et al., 1990; Dovrou et al., 2019; Froyd et al., 2010; Lee et al., 2003; Neubauer et al., 1996; Whiteaker & Prather, 2003).

Reaction 2 represents the equilibrium between aqueous phase formation and decomposition of HMS. A key question is whether decomposition following sample collection may have also contributed to HMS being overlooked in the observational record. For IC measurements, aerosol samples are typically collected on filters that are then extracted into solution; both the filters and solution extracts are often stored for some time before analysis. For example, in the Interagency Monitoring of Protected Visual Environments Network (IMPROVE), 1–2 months may elapse between sample collection and analysis (Solomon et al., 2014). This issue of potential HMS decomposition is similar to the problem of potential chemical transformations of secondary organic aerosol species occurring after sample collection (Eugene & Guzman, 2017; Rincón et al., 2009; Xia et al., 2018).

The fate of sampled HMS during the time interval of storage is unknown. If the aerosol particles in the filter samples dry out, and are kept in the dark, then any HMS present should be stable as a salt given sufficient cations. On the other hand, if the aerosol particles on the filters remain in aqueous phase, then HMS decomposition may occur via equilibrium reaction 2. HMS decomposition may also occur after extraction into solution. Work conducted in Beijing found that ~20% of the sulfur in a 100 μM HMS solution was oxidized to sulfate when the solution was extracted in water and allowed to sit for 80 min (Ma et al., 2020). For HMS in aqueous particles and extracted in solution, the pH would affect both equilibrium concentrations as well as the speed at which equilibrium is reached. In open systems, HMS decomposition would be expected to continue until a new equilibrium is reached with ambient SO_2_ and HCHO, both of which may be less abundant than under collection conditions. However, in closed systems at a given pH, HMS decomposition may be limited by a low air‐to‐solution volume ratio, which would lead to a smaller fraction of aqueous HMS decomposing to reestablish equilibrium in the system. Products of HMS decomposition may either oxidize to form sulfate or outgas from solution as HCHO and SO_2_. In all cases, the kinetics for aqueous HMS decomposition are slow but get faster with increasing pH; previous work has inferred an HMS lifetime in cloud or aerosol droplets on the order of months at pH 4.0, about 1 week at pH 5.0, and 1 day to only a few hours at pH 6.0 (Moch et al., 2018; Munger et al., 1986).

Recent studies have renewed interest in HMS as a significant contributor to particulate sulfur in Beijing, where high levels of particulate sulfur may be a key driver of heavy haze (Wang et al., 2014). In a 1‐D model study, the presence of HMS produced in clouds could explain the observed high levels of particulate sulfur during the extreme haze event in Beijing in January 2013 (Moch et al., 2018). A reanalysis of AMS observations in Beijing and new IC measurements have also suggested the likely presence of HMS there in winter (Ma et al., 2020; Song et al., 2019). HMS has also recently been detected in field campaigns in the Po Valley, Italy (Gilardoni et al., 2016; Paglione et al., 2020), and previously in the United Kingdom, Germany, Japan, and the United States (Dall'Osto et al., 2009; Dixon & Aasen, 1999; Lee et al., 2003; Scheinhardt et al., 2014; Suzuki et al., 2001; Whiteaker & Prather, 2003).

Here we build on these regional studies of HMS by examining the potential importance of HMS globally. We present new measurements and reassess old ones; we also test the decomposition of HMS via experiments by adding HMS in solution to filter samples in the laboratory. We compare the observations against results from the global chemical transport model GEOS‐Chem, updated with HMS chemistry. Lastly, we investigate the implications for pollution control by examining the controlling factors for HMS formation.

## 2 Data and Methods

### 2.1 GEOS‐Chem Model

To examine the importance of HMS for global particulate sulfur, we use GEOS‐Chem version 12.2.1 with detailed tropospheric chemistry (https://doi.org/10.5281/zenodo.2580198; Alexander et al., 2012; Park et al., 2004; Pye et al., 2009; Sherwen et al., 2016; Travis et al., 2016). The model is driven by assimilated meteorology from the NASA Modern‐Era Retrospective Analysis for Research and Applications, version 2 (MERRA‐2, Gelaro et al., 2017, https://gmao.gsfc.nasa.gov/reanalysis/MERRA-2/) at a global horizontal resolution of 2° × 2.5° for 6 years, 2013–2018, which helps account for interannual variability in cloud cover. To better capture the spatial variability over China, we also conduct nested simulations over East Asia at 0.5° × 0.625° horizontal resolution for January 2019, with boundary conditions taken from the global simulation and updated every 3 hr. Transport and convection in the model rely on a 10‐min time step, while chemistry and emissions use a 15‐min time step. Global anthropogenic emissions are from the Community Emissions Data System (CEDS, Hoesly et al., 2018), while biogenic volatile organic compound (VOC) emissions are from the Model of Emissions of Gases and Aerosols from Nature (MEGAN, Guenther et al., 2012) and biomass burning emissions from the Global Fire Emissions Database version 4 (GFED4, van der Werf et al., 2017). The CEDS inventory is overwritten by regional anthropogenic emissions inventories for Europe (www.ceip.at/), Canada (Environment and Climate Change Canada, 2018), the United States (Travis et al., 2016), Asia (Li, Zhang, et al., 2017), Africa (Marais & Wiedinmyer, 2016), and China (Zheng et al., 2018).

The gas phase and aqueous species partitioning of sulfur dioxide in clouds to form sulfite and bisulfite is included in the standard GEOS‐Chem. Here we implement gas phase and aqueous species partitioning for HCHO and HMS chemistry in clouds according to reactions 1 and 2, following Moch et al. (2018). These reaction rates are carried out in parallel with other sulfur chemistry in order to account for SO_2_ titration. After a cloud evaporates we assume HMS remains in aerosol and is chemically inert due to sufficiently acidic conditions. HMS in aerosol can later dissolve back into cloud water and become chemically active again. We assume that HMS has the same optical and deposition properties as sulfate. For the oxidation of HMS in clouds by aqueous hydroxyl radicals, we treat reaction 3 as the rate‐limiting step for the radical chain that terminates with the production of sulfate, and we collapse the chain following the dominant pathways suggested by Jacob (1986):

(4) HOCH2SO3−+OH·→SO2,HO2·2SO42−+HCHOaq+3H+

For the cloud water concentration of OH, we use a simple parameterization in which the ratio between aqueous and gas phase OH is 1.0 × 10^−19^ M cm^3^ molecule^−1^ (Jacob et al., 2005). In addition to HMS chemistry, our simulation also includes sulfur autoxidation in clouds catalyzed by transition metal ions (Shao et al., 2019).

Calculation of cloud pH through ion balance and the electroneutrality equation was introduced in GEOS‐Chem by Alexander et al. (2012). Here we have improved the stability of the solution by using Newton's method, and we have added aqueous phase formate, acetate, and Ca^2+^ and Mg^2+^ from dust to the ion balance. For Ca^2+^ and Mg^2+^ we assume that dust is 3.0% soluble calcium and 0.60% soluble magnesium by mass, as in Fairlie et al. (2010). Within the North China Plain area (33–43°N, 112–122°E), we constrain cloud pH to have a minimum value of 5 in order to better match observed values (Jiang et al., 2009; Li et al., 2018; Li, Wang, et al., 2017). Luo et al. (2020) give further details on how the revised cloud pH calculation improves the match between observed and simulated surface aerosol concentrations.

### 2.2 Measurement Sites and Techniques

We attempted to identify HMS in a set of chromatograms previously generated by analysis of samples from the IMPROVE network of aerosol composition in remote air (Solomon et al., 2014, http://vista.cira.colostate.edu/Improve/). IMPROVE samples were collected every 3 days on nylon filters and later analyzed by IC using the AS12A column (RTI International, 2016), a column type which has been shown to efficiently separate HMS and sulfate in the laboratory (Dovrou et al., 2019; Moch et al., 2018; Figure S1). Typically 1–2 months elapse between sample collection and sample analysis. During this period, the samples are shipped at ambient temperatures, first to a central cataloging facility and then to the analysis facility, where they are stored at <0°C in a filter cassette according to protocol. After extraction with deionized water and IC analysis, the samples are stored at 5.0°C (RTI International, 2016). The IMPROVE samples assessed here originate mainly from 156 sites in the United States, but also from one site in South Korea and one site in Canada.

We used the sodium salt of HMS (Alfa Aesar, 95% purity) to create a 1.0 ppm HMS solution (~55 μM) for a standard AS12A chromatogram against which to compare the IMPROVE chromatograms. The standard chromatogram revealed two peaks: the larger peak, representing ~75% of total peak area, corresponds to HMS, and the other peak corresponds to sulfate. The presence of the second peak indicates rapid HMS decomposition and oxidation to form sulfate under the standard IMPROVE measurement conditions, in which the pH of the eluent is typically ~8.0–9.0. By referring to the relative retention time between the HMS and sulfate peaks in the standard, we could then identify the presence of HMS in the IMPROVE sample chromatograms (Figure S1). We visually inspected 7,080 chromatograms from samples collected between August 2017 and October 2018 and analyzed by IC between October 2017 and December 2018. The set of chromatograms we examined do not span all IMPROVE sites nor include all samples over the time period from any single site. Importantly, we know from the observation of sulfate in the IC calibration runs of the HMS standard for the pH 8.0–9.0 eluent that HMS likely decomposes to some extent either in the IC under these conditions of high pH or during sample preparation. In addition, HMS may have decomposed in the IMPROVE samples even before analysis. For these reasons, we did not attempt to quantify the HMS in the IMPROVE data set; we merely recorded evidence of its presence.

Using IC, we also looked for evidence of HMS in new aerosol observations from China, Singapore, and Italy. In China, we collected PM_2.5_ filter samples in 12–18 January 2019, in Shijiazhuang, a city ~165 miles southwest of Beijing (Xie et al., 2019). One quarter of each quartz membrane filter was analyzed via IC using the AS14 column with an eluent pH of 9.0–10 on 21 January 2019, with HMS detected on six of the 14 filters. We also collected 17 samples in Singapore on borosilicate glass fiber filters over the time period March to December 2018. The Singapore samples were later shipped to Harvard University in Cambridge, USA, where they were analyzed in October–November 2019, using the AS12A column with an eluent of pH 7.0. Calibrations showed no appreciable decomposition of HMS at this pH in this IC setup. For our analysis, we extracted the material on one quarter of each of the filters in 20 mL milli‐Q water and sonicated this for 30 min at 25°C. Observations in Italy come from Bologna and from San Pietro Capofiume, located in the Po Valley (Paglione et al., 2020). Samples from Bologna were collected on 4–15 February and 7–18 October 2013, and on 3–21 February 2014. In San Pietro Capofiume, samples were collected only on 7–18 October 2013. All the samples from Italy were stored for 4–12 months and then dried and analyzed by NMR (Decesari et al., 2000; Gilardoni et al., 2016; Suzuki et al., 2001). Aerosol samples at the two Italy sites were also analyzed by IC using an AS11 column with KOH eluent, which cannot efficiently separate HMS and sulfate without special modifications to the operating procedure (Dovrou et al., 2019; Ma et al., 2020; Moch et al., 2018). Further details on observational set ups can be found in the supporting information Table S1.

Finally, we include in our analysis the observations previously reported from sites in Beijing, the United Kingdom, Germany, Japan, and the United States (Dall'Osto et al., 2009; Dixon & Aasen, 1999; Lee et al., 2003; Ma et al., 2020; Scheinhardt et al., 2014; Song et al., 2019; Suzuki et al., 2001; Whiteaker & Prather, 2003).

### 2.3 Decomposition Experiments

After the initial analysis, the Shijiazhuang filters were stored at −18°C. Roughly 4 months later, on 5 June 2019, we reanalyzed a second quarter of each of the six filters that had previously shown the presence of HMS. The reanalysis was performed in China via the same procedure as before.

In Cambridge, HMS decomposition was examined under laboratory conditions using an IC system (Dovrou et al., 2019). The IC calibration curves for HMS and sulfate in this system are shown in Figures S2 and S3. Two types of filters commonly used during field measurements, borosilicate glass fiber and nylon, were used for the decomposition tests. The borosilicate glass fiber filters had been previously used to sample ambient air in Singapore during March to December 2018. The nylon filters, the same type as those used in IMPROVE, were blank. The second quarter of each of the Singapore filters was spiked with 50 μL HMS solution of either 2.0 mM (0.10 μmol) or 2.0 M (100 μmol) created from a sodium‐HMS salt (Sigma‐Aldrich, 95% purity). Once the solution had been fully absorbed, these filters were stored at one of three temperatures: 25°C, 4°C, and −18°C. After time intervals ranging from 30 min to 2 months, the material on these filter quarters was extracted and analyzed via IC. A third, unspiked quarter of each Singapore filter was also stored at each of the three temperatures, and then analyzed via IC along with the spiked quarter. We applied the same spiking procedure to the blank nylon filters. The spiking method resulted in an extraction efficiency for the HMS solution of 18–30% for borosilicate glass fiber filters and of 14–20% for the nylon filters.

To determine the stability of HMS in solution in a closed system, the extract solutions made from the first, unspiked quarters of the Singapore filters were stored at 4.0°C over time intervals of 30 min to 2 months and then analyzed via IC. We also spiked a fourth quarter of each of the Singapore and the nylon filters, and extracts derived from these quarters were stored and analyzed via the same procedure.

## 3 Results

**Figure 1:**
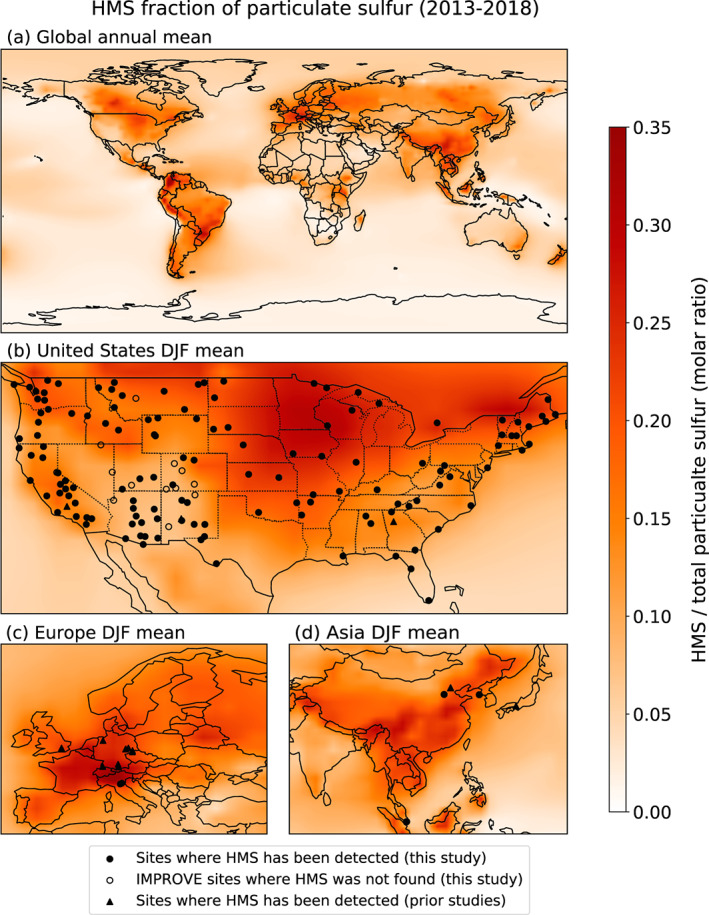
Mean molar fraction of HMS in total particulate sulfur (sulfate + HMS) at the surface calculated by GEOS‐Chem for 2013–2018 for the (a) global annual mean and the December‐January‐February (DJF) mean over (b) the United States, (c) Europe, and (d) Asia. Black dots indicate sites where at least trace amounts of HMS have been detected in new observations from the IMPROVE network; Shijiazhuang, China; Po Valley, Italy; and Singapore. Triangles indicate sites where HMS has previously been observed: Germany (Scheinhardt et al., 2014), the United Kingdom (Dall'Osto et al., 2009), the United States (Dixon & Aasen, 1999; Lee et al., 2003; Whiteaker & Prather, 2003), Japan (Suzuki et al., 2001), and Beijing (Ma et al., 2020; Song et al., 2019). Open circles indicate IMPROVE sites from which at least one chromatogram was examined but where HMS was not found.

We find evidence of HMS in observations from 160 locations across North America, Europe, and Asia (Figure 1). The vast majority of these sites are in the IMPROVE network, with at least trace amounts of HMS indicated by unquantified peaks similar to those shown in Figure S1. Altogether, evidence of HMS appears at 139 of the 158 IMPROVE sites worldwide, including sites in the United States, South Korea, and Canada. Of the 7,085 IMPROVE chromatograms examined, 1,888 (~27%) reveal a peak with an elution time corresponding to that of HMS. Some HMS in the IMPROVE samples may have decomposed in the time between sample collection and analysis or, as suggested by the sulfate peak in the HMS standard, even during analysis. The extent of such decomposition is unknown. As is discussed below, we also detect and attempt to quantify the presence of HMS in samples from Singapore; the Po Valley, Italy; and Shijiazhuang, China. The rest of the sites with observational evidence of HMS shown in Figure 1 are from previous reports at 17 sites including nine in Germany (Scheinhardt et al., 2014), one in the United Kingdom (Dall'Osto et al., 2009), four in the United States (Dixon & Aasen, 1999; Lee et al., 2003; Whiteaker & Prather, 2003), one in Japan (Suzuki et al., 2001), and two in Beijing (Ma et al., 2020; Song et al., 2019).

In GEOS‐Chem, the global mean fraction of HMS in particulate sulfur is ~5–6% in surface air over 2013–2018, with a mean fraction over land of ~10%. For many regions, the model yields seasonal HMS fractions of 25% or more of particulate sulfur (Figures 1 and S4, Table S2). Over North America, Europe, and China, the modeled HMS fraction of particulate sulfur is largest in boreal winter (DJF) relative to other seasons. More specifically, HMS accounts for 20–30% of particulate sulfur across the northern United States, southern Canada, and much of Europe and China at that time of year. In June‐July‐August (JJA), HMS as a fraction of particulate sulfur is at a minimum over the United States and Europe, but at a maximum over India, where the mean fraction of HMS is nearly 20%. Also in JJA, HMS exceeds 25% of total particulate sulfur over northern Canada and Alaska, as well as over much of South America. For March‐April‐May (MAM), HMS makes up ~30% of particulate sulfur for the northern and central United States. Across all seasons, HMS fraction ranges from ~7% to 30% of particulate sulfur over most of the United States.

**Figure 2:**
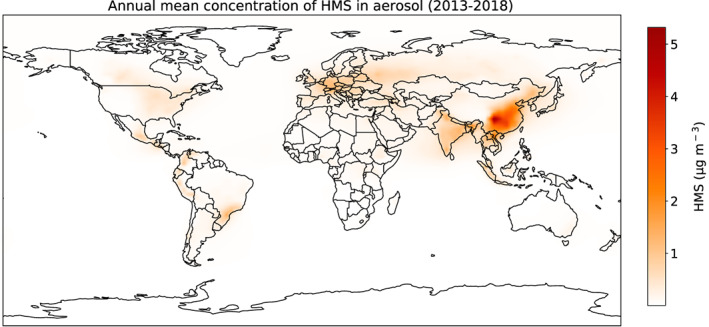
Annual mean mass concentration of HMS at the surface, calculated by GEOS‐Chem for 2013–2018.

Figure 2 shows annual mean HMS mass concentrations for 2013–2018. Annual mean simulated HMS mass is highest in China, with a maximum of ~5.0 μg m^−3^ in the Sichuan Basin area. Annual means in excess of 1.0 μg m^−3^ are also found in central and eastern Europe, India, Southeast Asia, Mexico, southern Brazil, and the northern Andes. Seasonal mean HMS mass concentrations are usually highest during continental winter (Figure S5, Table S2).

The spatial patterns of modeled HMS are influenced by variations in temperature, cloud water content, cloud pH, and HCHO and SO_2_ concentrations. The sources and sinks of HMS accordingly vary seasonally, with wet deposition acting as the predominant sink responsible for nearly 80% of HMS removal (Table S3). Areas of high absolute HMS concentration generally have abundant HCHO and SO_2_ and frequent clouds. Some remote regions, such as in the Andes, also exhibit significant HMS due to abundant cloud water and adequate HCHO to compete with photochemical oxidants for SO_2_. Several factors drive the modeled seasonality of HMS concentrations and the HMS fraction of particulate sulfur. At a given concentration of HCHO and SO_2_, colder temperatures increase the solubility of HCHO and SO_2_ and thus drive an overall increase in the net HMS formation rate despite the reduction in the rate constant for this reaction. Reduced sunlight, often associated with colder temperatures, generally means fewer oxidants are available to oxidize HMS or to compete with HMS formation from SO_2_ and HCHO, which in turn decreases sulfate abundance while increasing HMS. However, both colder temperatures and reduced sunlight can also diminish biogenic VOC emissions and slow oxidation of VOCs from any source to form HCHO, yielding lower HCHO concentrations overall, even as the HCHO lifetime lengthens. Concentrations of SO_2_ can also vary seasonally; in regions where coal is an important source of residential heating, SO_2_ emissions generally peak during winter. The net effect of these seasonal influences is generally to promote higher HMS in winter, both in terms of concentration and fraction of particulate sulfur.

**Figure 3:**
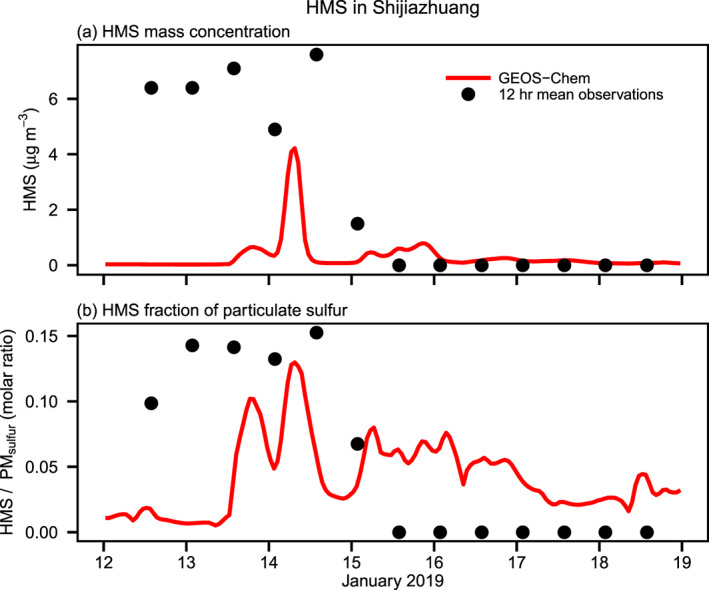
Observed and simulated (a) HMS concentrations and (b) molar fraction of HMS in total particulate sulfur (sulfate + HMS) at the surface during 12–19 January 2019, in Shijiazhuang, China. The red line indicates hourly model results from GEOS‐Chem at 0.5° × 0.625° resolution for Shijiazhuang, and the black dots represent 12‐hr mean observations in Shijiazhuang made via ion chromatography with an AS14 column, which can distinguish HMS from sulfate. Samples from Shijiazhuang were stored for approximately 1 week between collection and analysis.

In Shijiazhuang, China, the samples collected on 12–19 January 2019 and analyzed on 21 January show a maximum HMS concentration of 7.6 μg m^−3^ and a mean of 2.5 μg m^−3^ (Figure 3). GEOS‐Chem during this timeframe simulates a maximum of ~4.0 μg m^−3^ HMS with a mean of only 0.27 μg m^−3^. While the absolute concentrations of HMS are underestimated by GEOS‐Chem, the ratio of HMS to sulfate in PM agrees better with observations. In observations, the maximum HMS to total particulate sulfur ratio is ~15% with a mean of 5%, while GEOS‐Chem yields a maximum of ~11% and a mean of 3% (Figure 3). Six of the 14 Shijiazhuang filters revealed the presence of HMS when analyzed on 21 January; after 4 months storage these six filters showed only trace levels of HMS remaining, similar to the small peaks seen in IMPROVE (Figure S1).

HMS abundance in Shijiazhuang is variable in both the observations and GEOS‐Chem, with concentrations dropping to near zero after 15 January in the model and no HMS detected after the morning of 15 January. Figure 3 shows hourly averages from GEOS‐Chem in order to illustrate the strong temporal variability in modeled HMS concentrations, which is due to the episodic presence of low clouds (Moch et al., 2018). With the presence of low clouds in a GEOS‐Chem gird box, HMS is quickly produced via reactions 1 and 2. Once the clouds dissipate, however, HMS is either transported away or deposited at the surface, and local HMS levels drop. If HMS is transported to an environment lower in SO_2_ and HCHO than the source region, the aqueous phase chemistry shifts the equilibrium of reaction 2, driving destruction of HMS at rates that increase with increasing cloud pH, which is typically in the range of ~3–8 depending on the region (Shah et al., 2020). During haze events in China, India, and other polluted regions, HMS may account for a much larger percentage of particulate sulfur than is indicated by the seasonal averages (Figures 1 and S4), with HMS nearing 50% of particulate sulfur during some events. As an example, Figure S6 shows observations and model results for a haze event in January 2013 in Beijing, previously analyzed with a 1‐D model in combination with GEOS‐Chem in Moch et al. (2018) and revisited here using GEOS‐Chem with HMS chemistry included within the model.

All observations from Singapore reveal a significant presence of HMS. Between 12 March 2018, and 22 December 2018, HMS concentrations occur within a relatively narrow range of 0.50 to 0.63 μg m^−3^, with a mean of 0.57 μg m^−3^ (Figure S7). In GEOS‐Chem, the monthly mean values of HMS concentration are generally lower than the daily observations, except during JJA, when the model matches observations within ~0.10 μg m^−3^ (Figure S7). In contrast to Shijiazhuang, no HMS decomposition was observed in the Singapore samples between September and November 2019 at the laboratory in Cambridge.

For the Po Valley, Italy, HMS was detected via NMR in 93% of the samples, with concentrations varying from ~2.0 to 230 ng m^−3^, or 1–10% of total particulate sulfur. As with the IMPROVE samples, it is not known to what extent HMS may have decomposed during storage or analysis. Whether HMS decomposed during the drying period prior to NMR analysis is also unknown. In any event, these observations suggest a mean of 0.05 μg m^−3^ in February 2014 in this region, accounting for 4% of total particulate sulfur, while GEOS‐Chem calculates a mean of 0.52 μg m^−3^, equivalent to ~20% of particulate sulfur (Figure S8). The GEOS‐Chem monthly mean values of HMS concentration and HMS as a fraction of particulate sulfur in the Po Valley are also higher than any of the observations reported here (Figures S8 and S9).

In the spiking experiments, water from the HMS solutions was visibly absorbed into both the borosilicate glass fiber and nylon filters, leaving the solution droplets to dry out on the filter surface. For the spiking experiments using a 2 M HMS solution on borosilicate glass fiber filters, subsequent IC analysis showed a significantly enhanced concentration of sulfate, equivalent to ~40% of the additional sulfur detected in the spiked samples, along with the higher levels of HMS (Figure S10). The subsequent IC analyses across all spiked samples revealed that the HMS concentrations remained roughly constant or declined slightly during the 30‐min to 2‐month periods under which the samples were monitored (Figures S10 and S11). The slight declines in HMS concentration, on the order of ~10–15%, were generally accompanied by a similar or greater percentage decline in concentrations of sulfate, suggesting these changes were due to variability in extraction efficiency (Figure S10).

**Figure 4:**
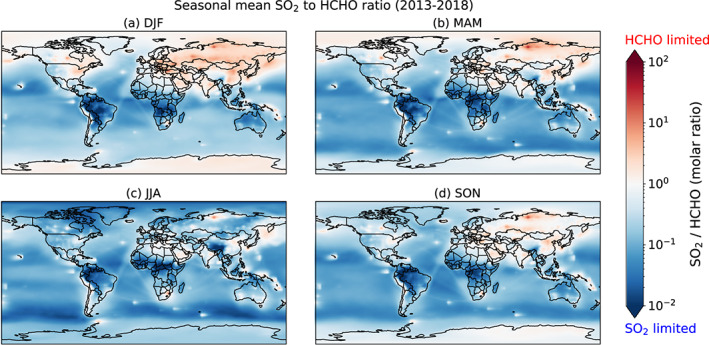
Seasonal mean molar ratio of SO_2_ to HCHO in air below ~1 km above the surface for 2013–2018. Red colors indicate higher levels of SO_2_ compared to HCHO and therefore generally HCHO‐limited conditions for HMS formation. Blue colors indicate higher levels of HCHO compared to SO_2_ and therefore generally SO_2_‐limited conditions. Here we make the assumption that the SO_2_ and HCHO lifetimes against processes other than HMS formation are longer than an hour, which is the typical lifetime for SO_2_ and HCHO against conversion to HMS in clouds.

The potentially large contribution of HMS to particulate sulfur has important implications for pollution control strategies. As shown in reactions 1 and 2, sulfite or bisulfite and HCHO react with a 1:1 ratio to form HMS. A typical lifetime for SO_2_ and HCHO against conversion to HMS in low clouds is less than 1 hr (Moch et al., 2018). Here we assume that the SO_2_ and HCHO lifetimes against processes other than HMS formation are longer than an hour. This will almost always be the case for SO_2_ (Lee et al., 2011) and will usually be the case for HCHO, especially during winter when oxidants and sunlight are low (Anderson et al., 2017; Zhu et al., 2016). Both molecules can then be said to deplete at the same rate in HMS formation, and thus each can act as the limiting reactant (Moch et al., 2018). Figure 4 shows the seasonal mean ratio of SO_2_ to HCHO for air from the surface to ~1 km for 2013–2018. Ratios greater than 1.0 imply that HMS formation is HCHO‐limited and ratios less than 1.0 imply SO_2_ limitation. The SO_2_‐to‐HCHO ratio shows much spatial heterogeneity that varies by season, but a few patterns emerge. In Northern Hemisphere winter, HMS is HCHO‐limited for much of Asia and Europe, although the Po Valley in Italy, an HMS hotspot in our simulation, is slightly SO_2_‐limited at this time of year. The eastern United States is also HCHO‐limited during DJF but is generally SO_2_‐limited along with the rest of the country during other seasons. The North China Plain is HCHO‐limited year‐round but has the weakest HCHO limitation in JJA. Southeast Asia along with most of South America is SO_2_‐limited year‐round. Finally, India is HCHO‐limited during all seasons except JJA, when it is SO_2_‐limited in some areas and HCHO‐limited in others. As described previously, HCHO and SO_2_ can vary seasonally and regionally due to emissions and the concentrations of oxidants, and these variations drive the spatial patterns in the SO_2_‐to‐HCHO ratio. For example, eastern China experiences higher SO_2_ emissions during DJF due to additional coal burning, especially for residential heating. At the same time, lower oxidant levels during DJF further increase SO_2_ by decreasing the efficiency at which SO_2_ is converted to sulfate. The low oxidant levels in winter also slow the formation rate of secondary HCHO from VOCs.

## 4 Discussion and Conclusion

GEOS‐Chem simulations suggest that HMS may comprise a quarter or more of seasonal mean particulate sulfur in multiple polluted regions, with even higher percentages possible during pollution episodes. Simulated HMS is generally highest in continental winter, both in terms of absolute values (as much as ~7.0 μg m^−3^) and as a percentage of particulate sulfur (as much as 30–35%). Simulated HMS is roughly consistent with quantitative observations from Shijiazhuang, China, and from Singapore, which reveal substantial amounts of HMS. A global presence of HMS is also supported by IMPROVE data, which show the ubiquitous presence of at least trace amounts of HMS throughout the United States across all seasons. Further evidence for at least trace amounts of HMS comes from the Po Valley, Italy.

The time elapsed between the sample collection and the analysis varies among the measurements discussed in this work as do the details of the analytical approaches, and the laboratory experiments on HMS stability are inconclusive. Neither the spiked samples on borosilicate glass fiber and nylon filters nor the HMS extracts in closed systems examined in this work clearly show major HMS decomposition over time intervals ranging from 30 min to 2 months. Observations from Singapore are consistent with this, with HMS on borosilicate glass fiber filters surviving for over a year at concentrations of 100 s of ng m^−3^, with no significant decomposition detected over 2 months of consistent monitoring. In contrast, in the Shijiazhuang samples, HMS on the quartz membrane filters decomposed from as much as 7.6 μg m^−3^ to just trace amounts after 4 months of storage. Borosilicate glass fiber filters from Singapore spiked with 2 M HMS solution showed significantly enhanced concentrations of sulfate, suggesting a large portion of the HMS solution quickly decomposed and oxidized to sulfate under those conditions. In the IMPROVE IC system, a sulfate peak appeared immediately in the chromatograph of the pure HMS standard, suggesting decomposition in that system either in solution or from the eluent, which typically had a pH of 8.0–9.0.

The reasons for these contradictory results on decomposition are elusive. From a kinetic perspective, one would expect HMS to remain stable in solid, dry form, and this may explain the stability of the Singapore samples. Stable HMS concentrations would also be expected in the aqueous phase if equilibrium is achieved between HMS in solution and gas phase SO_2_ and HCHO within a closed system. The aerosol matrix, filter material, variability in humidity, temperature, particle acidity, presence of gases absorbed into the filter material, and (in closed systems) the amount of HMS may all influence whether or not HMS decomposition occurs during sample storage. The spiking experiments with HMS solution droplets may therefore be imperfect analogs for how HMS in aerosol behaves during storage. In addition, variability in extraction efficiency increases the uncertainty among data points obtained during the spiking experiments. If HMS decomposition does occur, the relative fraction of the resulting sulfite that is oxidized to sulfate or outgases as SO_2_ will also likely vary depending on conditions, such as pH or the concentrations of HCHO and SO_2_. Given these issues we argue that rapid analysis of samples shortly after collection using methods capable of differentiating HMS and sulfate is the best way to determine HMS abundance. A particle‐into‐liquid sampler coupled with IC and single particle mass spectrometry coupled with high performance liquid chromatography are two examples of systems that could fit these criteria.

Since some measurement methods can easily misinterpret HMS as sulfate, validation of the model results with observations is challenging. The issue of possible HMS decomposition also complicates model validation. Additionally, the cloud processing of HMS is likely characterized by fine‐scale spatial heterogeneity, while the model resolution used here is relatively coarse at 2° × 2.5° everywhere except over East Asia, where the resolution is 0.5° × 0.625°. Additional sources of error include uncertainty in the simulated cloud cover (Moch et al., 2018) as well as model underestimates in HCHO concentrations in certain regions during winter (Jaeglé et al., 2018; Rao et al., 2016; Figure S6). Nonetheless, the well‐established HMS chemistry in combination with the observations made to date suggest that HMS may be an important contributor to particulate sulfur. The large abundance of HMS measured at Shijiazhuang (nearly 8.0 μg m^−3^) provides compelling evidence of this contribution, and the relatively close match between the measured and modeled HMS to sulfate PM ratio at this site is reassuring.

AMS measurements in Beijing also suggest the presence of HMS at concentrations up to ~30% of particulate sulfur during haze events (Song et al., 2019). These authors attributed the production of high levels of HMS to aqueous phase reactions in aerosol water. Although HMS chemistry should occur in all aqueous media given sufficient HCHO and SO_2_, it seems likely that cloud chemistry plays a more important role in HMS formation, at least during cloudy periods, given the lower pH of aerosols and much smaller volume of aerosol water compared to cloud water which drive in‐aerosol HMS formation rates to orders of magnitude less than in‐cloud rates given the same HCHO and SO_2_ concentrations (Moch et al., 2018). The episodic nature of clouds could also help explain the variability of HMS concentrations seen in both the AMS measurements in Beijing and the IC measurements in Shijiazhuang.

Our results suggest that controlling HCHO can be an effective particulate sulfur control strategy in some regions, by reducing either the direct emissions of primary HCHO or the VOC emissions which lead to secondary HCHO. This in turn implies that addressing a different set of pollution sources than is typical may be required: Emissions of SO_2_ are dominated by coal (Smith et al., 2011), while anthropogenic primary and secondary HCHO are often derived from oil and gas and the transportation sector (Li, Zhang, et al., 2017; Zhu et al., 2017). However, the effectiveness of HCHO for controlling HMS varies by region and season. The possible role of HCHO in PM_2.5_ production is consistent with two instances in the observational record where SO_2_ reductions were not as effective at reducing pollution as anticipated. First, between 2013 and the beginning of 2017 in Beijing, the number of extreme haze days each winter, defined as days when the average PM_2.5_ of 12 monitoring sites across the city exceeded 200 μg m^−3^, did not significantly decline even as SO_2_ concentrations dropped by over 60% (Moch et al., 2018). Second, in the eastern United States, SO_2_ emissions fell precipitously by 68% between 2007 and 2015, but sulfate declined only 40% in winter compared to 72% in summer (Shah et al., 2018). These unexpectedly weak responses to reductions in SO_2_ emissions may be explained in part by limited availability of oxidants in winter. But the observations and results from GEOS‐Chem presented here suggest that HMS may account for a large fraction of particulate sulfur in both China and the eastern United States and that restricting SO_2_ emissions has therefore a smaller benefit for air quality than would be expected under the HCHO‐limited conditions in these regions in winter.

## Supporting information

Supporting Information S1

## Data Availability

Observational data are available online (https://doi.org/10.7910/DVN/TYGCJ8).
