# Supplementary material for: Global Importance of Hydroxymethanesulfonate in Ambient Particulate Matter: Implications for Air Quality
Source: J Geophys Res Atmos. 2020 Sep 11;125(18):e2020JD032706. doi: 10.1029/2020JD032706 (PMC7685164; doi:10.1029/2020JD032706)
Supplement: Supplementary file 1 — Supporting Information S1 [file JGRD-125-e2020JD032706-s001.pdf]

Journal of Geophysical Research – Atmospheres

Supporting Information for

Global importance of hydroxymethanesulfonate in ambient particulate matter: Implications for air quality

Jonathan M. Moch[1], Eleni Dovrou[2], Loretta J. Mickley[2], Frank N. Keutsch[1,2,3], Zirui Liu[4], Yuesi Wang[4], Tracy L. Dombek[5], Mikinori Kuwata[6†], Sri Hapsari Budisulistiorini[6,† †], Liudongqing Yang[6], Stefano Decesari[7], Marco Paglione[7], Becky Alexander[8], Jingyuan Shao[8,9], J. William Munger[1,2], Daniel J. Jacob[1,2]

[1] Department of Earth and Planetary Sciences, Harvard University, Cambridge, MA, USA
[2] John A. Paulson School of Engineering and Applied Sciences, Harvard University, Cambridge, MA, USA
[3] Department of Chemistry and Chemical Biology, Harvard University, Cambridge, MA, USA
[4] State Key Laboratory of Atmospheric Boundary Layer Physics and Atmospheric Chemistry, Institute of Atmospheric Physics, Chinese Academy of Sciences, Beijing, China
[5] Environmental Chemistry Division, RTI International, Research Triangle Park, NC, USA
[6] Asian School of the Environment and Earth Observatory of Singapore, Nanyang Technological University, Singapore
[7] Italian National Research Council - Institute of Atmospheric Sciences and Climate (CNR-ISAC), Bologna, Italy
[8] Department of Atmospheric Sciences, University of Washington, Seattle, Washington, USA.
[9] College of Flying Technology, Civil Aviation University of China, Tianjin, China
[†] Now in the Department of Atmospheric and Oceanic Sciences, School of Physics, and BIC-ESAT, Peking University, China
[††] Now in Wolfson Atmospheric Chemistry Laboratories, Department of Chemistry, University of York, UK

Contents of this file

Figure S1. Example IMPROVE chromatogram with HMS
Figure S2: HMS calibration curve at sample pH=5.5 and eluent pH=7 for IC system used for analysis of Singapore samples and for the decomposition experiments
Figure S3: Sulfate calibration curve at sample pH=5.5 and eluent pH=7 for IC system used for analysis of Singapore samples and for the decomposition experiments
Figure S4. Simulated seasonal mean molar fraction of HMS in total particulate sulfur for 2013-2018
Figure S5. Simulated seasonal mean mass concentration of HMS for 2013-2018
Figure S6. Timeseries of observed and simulated sulfate and total particulate sulfur for January 2013 in Beijing

Figure S7. Observed and simulated HMS concentrations for Singapore between March 2018 and December 2018
Figure S8. Observed and simulated HMS concentrations and molar fraction of HMS in particulate sulfur in February 2014 for Bologna, Italy
Figure S9. Observed and simulated HMS and sulfate concentrations for February 2013 to February 2014 in Bologna, Italy
Figure S10: Example results for HMS spiking experiments with borosilicate glass fiber filters from Singapore
Figure S11: Example results for HMS spiking experiments with blank nylon filters
Figure S12: Example chromatogram from the decomposition experiments conducted with the borosilicate glass fiber filters from Singapore
Tables S1. Comparison of measurement sites and techniques
Table S2. Simulated mean HMS concentrations and mean fraction of HMS in particular sulfur for the globe and selected regions for 2013-2018
Tables S3. Simulated global mean burden, sources, and sinks of HMS for 2017-2018

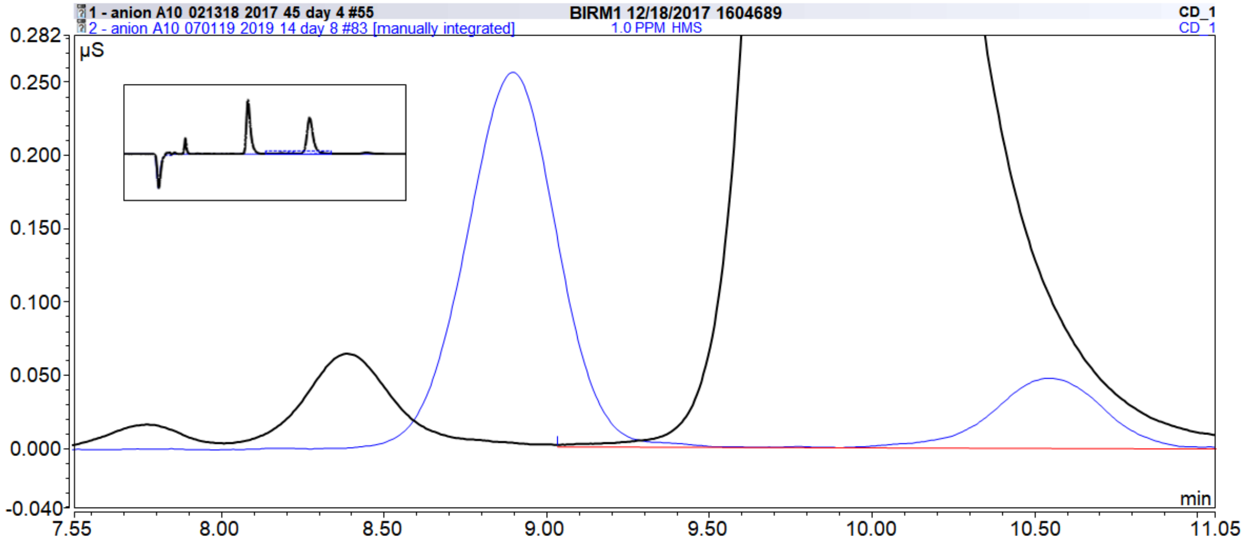

Figure S1: Example IMPROVE chromatogram from a sample collected on December 18, 2017, in Birmingham, Alabama, and analyzed with an AS12A column. The blue line represents the HMS standard, with the first peak corresponding to HMS and the second peak corresponding to sulfate generated from HMS decomposition, possibly due to the eluent pH of ~8-9. The black line shows the sample detection, with the peak centered at ~8.40 minutes corresponding to HMS and the large peak centered at ~10.05 minutes corresponding to sulfate. The peaks in the HMS standard as shown here are shifted ~0.5 minutes later than the Birmingham sample.

Table S1: Comparison of measurement sites and techniques

	IMPROVE	Shijiazhuang	Singapore	Po Valley
Site location	Various	38.03°N, 114.48°E	1.35°N, 103.68°E	44.52°N, 11.33°E and 44.65°N, 11.61°E
Site elevation	Various	27 m above ground	Roof of 5 story building	50 m (Bologna) and 10 m a.s.l. (S. Pietro Capofiume)
Surrounding area	Usually remote	Urban, commercial and residential area	Urban, near industrial area	Urban (Bologna) and rural (San Pietro Capofiume)
Sampling duration	24 hours beginning at 24:00 local time	11.5 hours, beginning at 8:00 or 20:00 local time	23 hours beginning at 9:00 or 10:00 local time	8-16 hours beginning at 9:00 or 17:00 local time
Filter material	Nylon	Quartz membrane	Borosilicate glass fiber (GB-100R)	Quartz fiber
Filter dimensions	37 mm	90 mm	47 mm	9-15cm diameter
Filter pore size	0.5 µm	N/A	0.3 µm	N/A
Filter manufacturer	Pall Corporation, USA	Pall Corporation, USA	Advantec, Japan	Pall Corporation, USA
Time between sampling and initial analysis	~1-2 months	~Days	10-19 months	4-12 months
Extraction method	Sonicated for 30 minutes after adding 20 mL DI water and allowed to sit overnight	25 mL of DI water for 30 minutes	20 mL of milli-Q water and sonicated for 30 minutes	Extracted in water
IC type	Dionex ICS-3000	Dionex ICS-90	Dionex ICS-5000+	Dionex ICS-2000 system
IC column	AS12A	AS14	AS12A	AS11
IC Flow rate	1.5 mL min^{-1}	1.0 mL min^{-1}	1.5 mL min^{-1}	0.25 mL min^{-1}
Eluent type and concentration	2.7 mM carbonate / 0.3 mM bicarbonate	3.5 mM carbonate / 1.0 mM bicarbonate	4.5 mM carbonate / 1.4 mM bicarbonate	0.1mM to 38 mM Potassium hydroxide
Eluent pH	~8-9	~9-10	7	~11-13

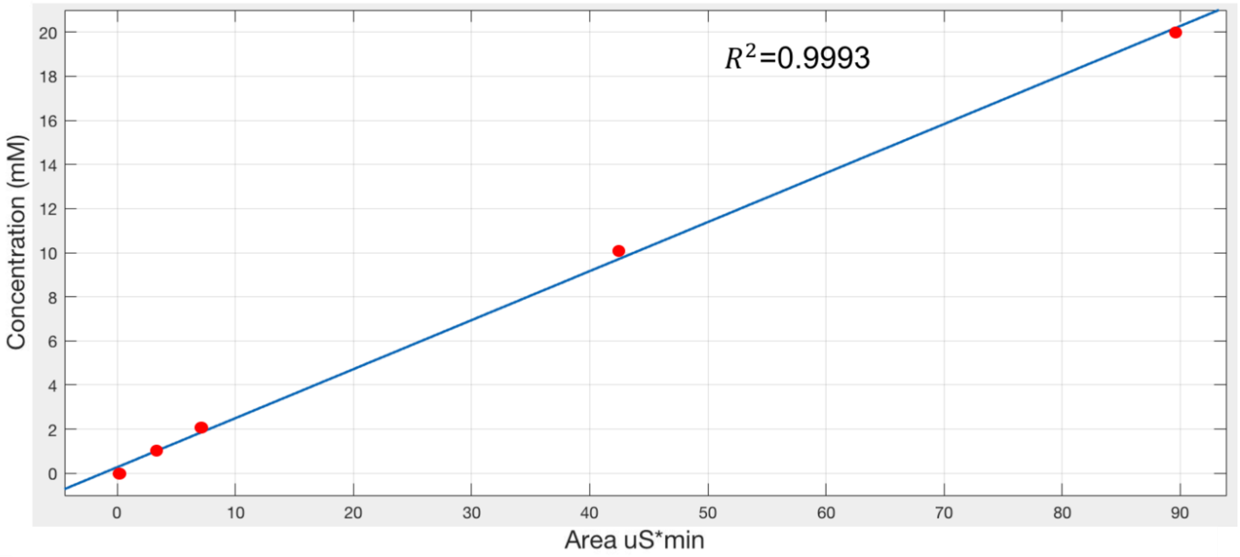

Figure S2: HMS calibration curve at sample pH=5.5 and eluent pH=7 for IC system used for analysis of Singapore samples and for the decomposition experiments.

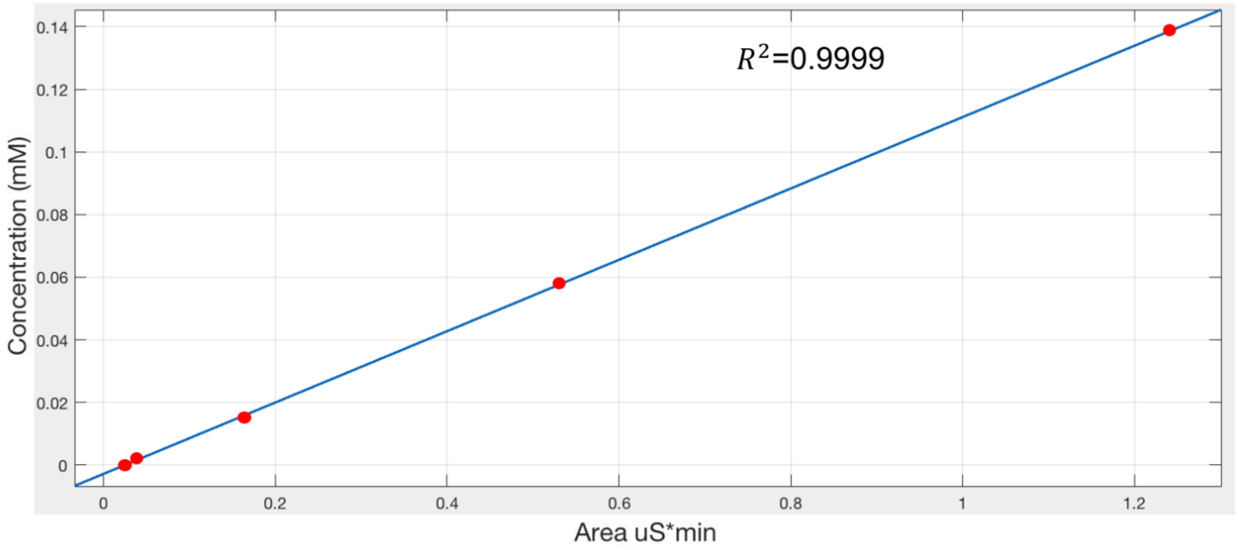

Figure S3: Sulfate calibration curve at sample pH=5.5 and eluent pH=7 for IC system used for analysis of Singapore samples and for the decomposition experiments.

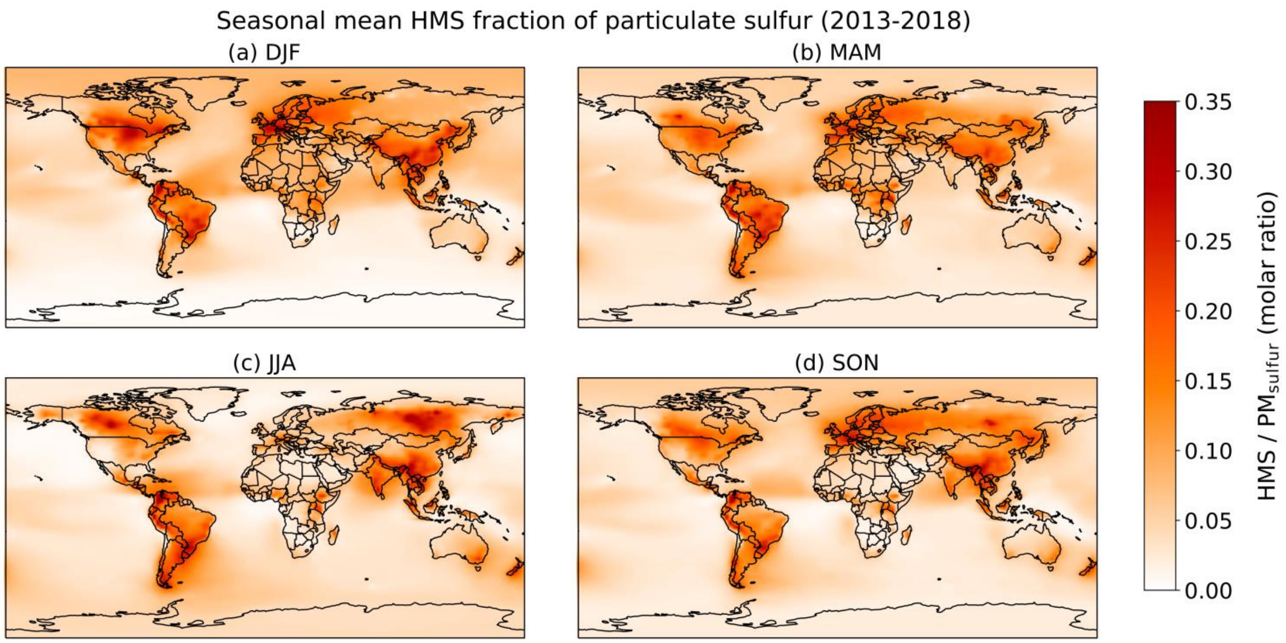

<u>Figure S4:</u> Seasonal mean molar fraction of HMS in total particulate sulfur (sulfate + HMS), simulated by GEOS-Chem for 2013-2018 during (a) December-January-February, (b) March-April-May, (c) June-July-August, and (d) September-October-November.

Table S2: Simulated mean HMS concentrations and mean fraction of HMS in particulate sulfur for the globe and selected regions for 2013-2018. Mean HMS concentrations are in units of μg m^{-3}, followed by the fraction of HMS in particulate sulfur in parentheses.

Region	Annual	DJF	MAM	JJA	SON
Global	0.095 (0.060)	0.12 (0.063)	0.096 (0.063)	0.077 (0.057)	0.092 (0.057)
Continental	0.24 (0.10)	0.28 (0.11)	0.23 (0.11)	0.20 (0.097)	0.24 (0.097)
United States	0.19 (0.12)	0.21 (0.15)	0.24 (0.14)	0.15 (0.069)	0.16 (0.11)
Europe	0.56 (0.16)	0.78 (0.20)	0.65 (0.18)	0.18 (0.079)	0.62 (0.18)
China	0.92 (0.16)	1.31 (0.19)	0.77 (0.16)	0.69 (0.15)	0.93 (0.16)
India	0.93 (0.13)	0.87 (0.12)	0.55 (0.079)	1.1 (0.19)	1.2 (0.15)

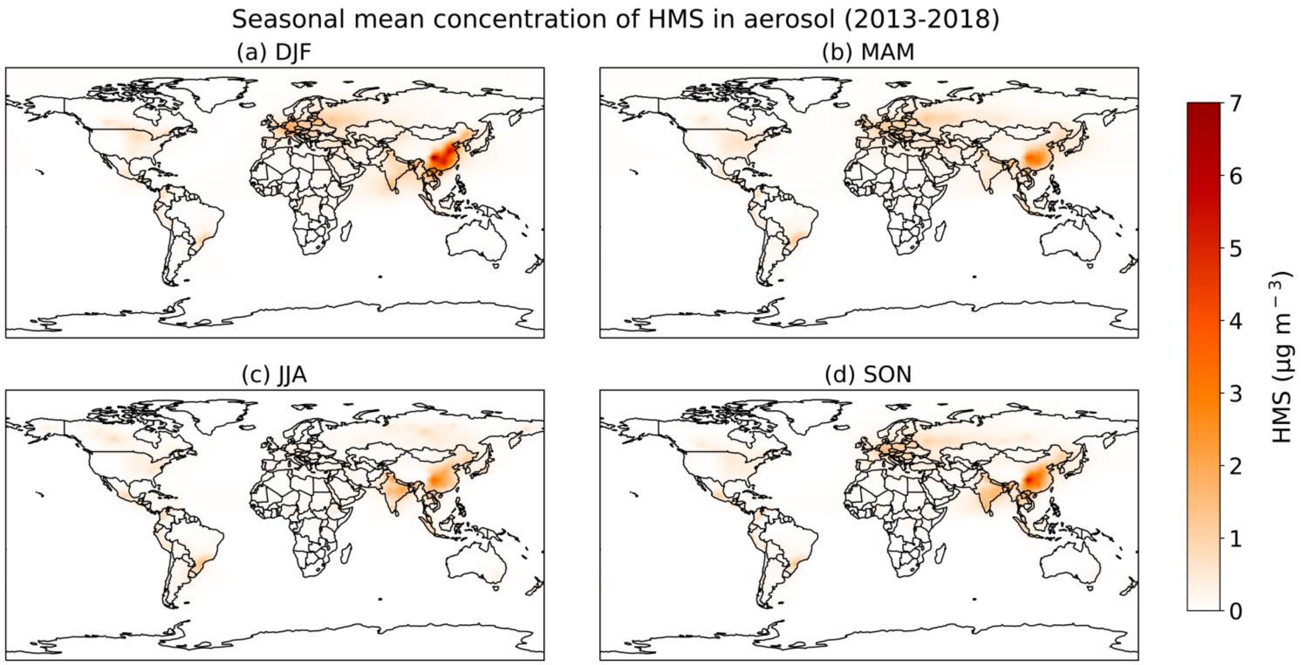

Figure S5: Seasonal mean mass concentrations of HMS simulated by GEOS-Chem for 2013-2018 during (a) December-January-February, (b) March-April-May, (c) June-July-August, and (d) September-October-November.

Table S3: Simulated global mean burden, sources, and sinks of HMS for 2017-2018. Global burden is in units of Gg sulfur and sources and sinks are in units of Gg sulfur per year or season.

	Annual	DJF	MAM	JJA	SON
HMS global burden	25.6	26.6	32.0	21.6	22.2
HMS production in cloud from SO_2 and HCHO	3110	717	844	827	718
HMS loss in cloud from reaction with OH^-	181	29.5	46.4	74.1	31.2
HMS loss from oxidation by OH to SO_4^{2-}	91.2	21.0	22.8	24.9	22.6
HMS wet deposition	2480	561	687	660	569
HMS dry deposition	388	110	101	81.6	95.4

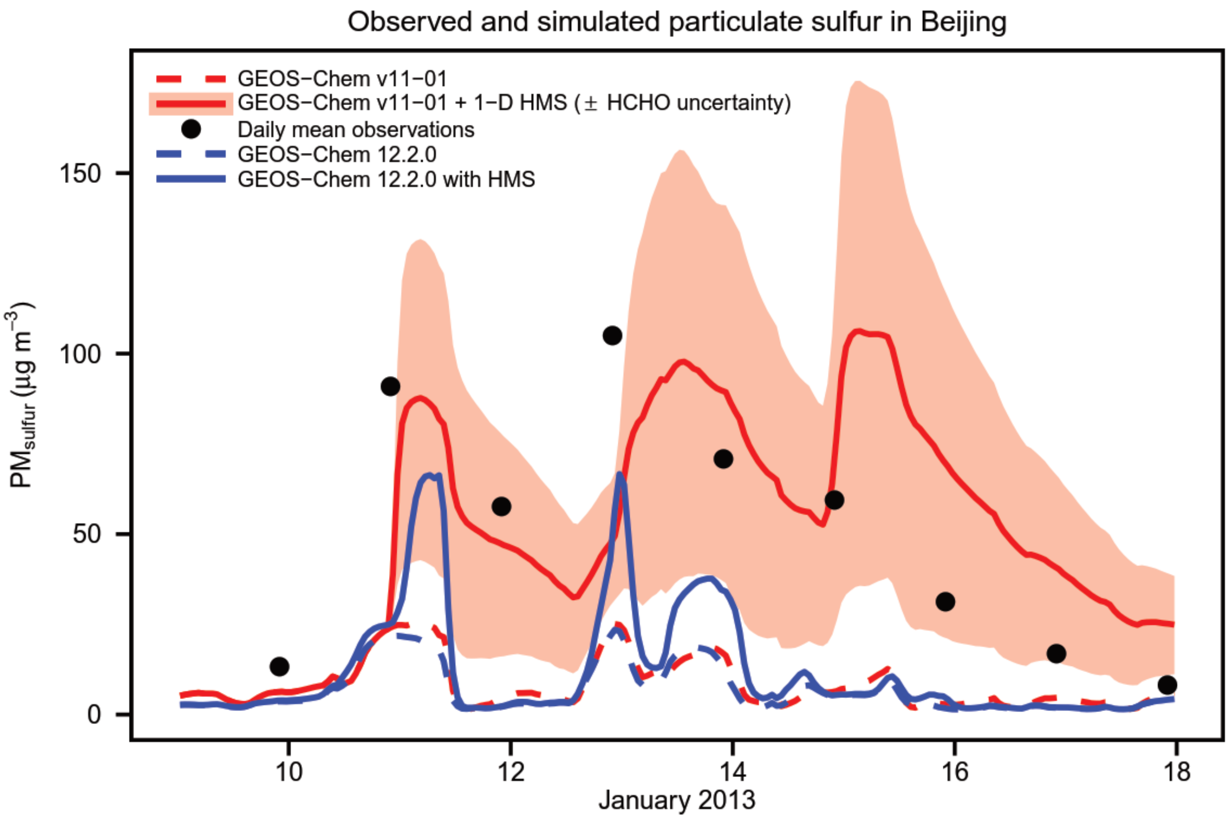

Figure S6: Timeseries of observed and simulated PM components for January 2013 in Beijing. The black points represent daily mean observed particulate sulfur concentrations measured at Tsinghua University, centered at 10 p.m. local time, or midway between the start and end times of the daily filter measurements (Cao et al., 2014). The red dashed line indicates hourly sulfate concentrations from GEOS-Chem version 11-01 and the solid red line represents the hourly sum of GEOS-Chem sulfate and HMS concentrations generated by the 1-D HMS model of Moch et al. (2018), assuming a mean HCHO concentration over the time period of 5.5 ppb. The pink shading indicates uncertainty in the 1-D model for a mean formaldehyde concentration between 1.6 and 9.4 ppb. Blue lines represent hourly concentrations from GEOS-Chem v12.2.0 with HMS chemistry (this work), with the dashed line denoting sulfate and the solid line representing the sum of HMS and sulfate concentrations. In GEOS-Chem v12.2.0, the mean local HCHO concentration over this time period is 2.4 ppb.

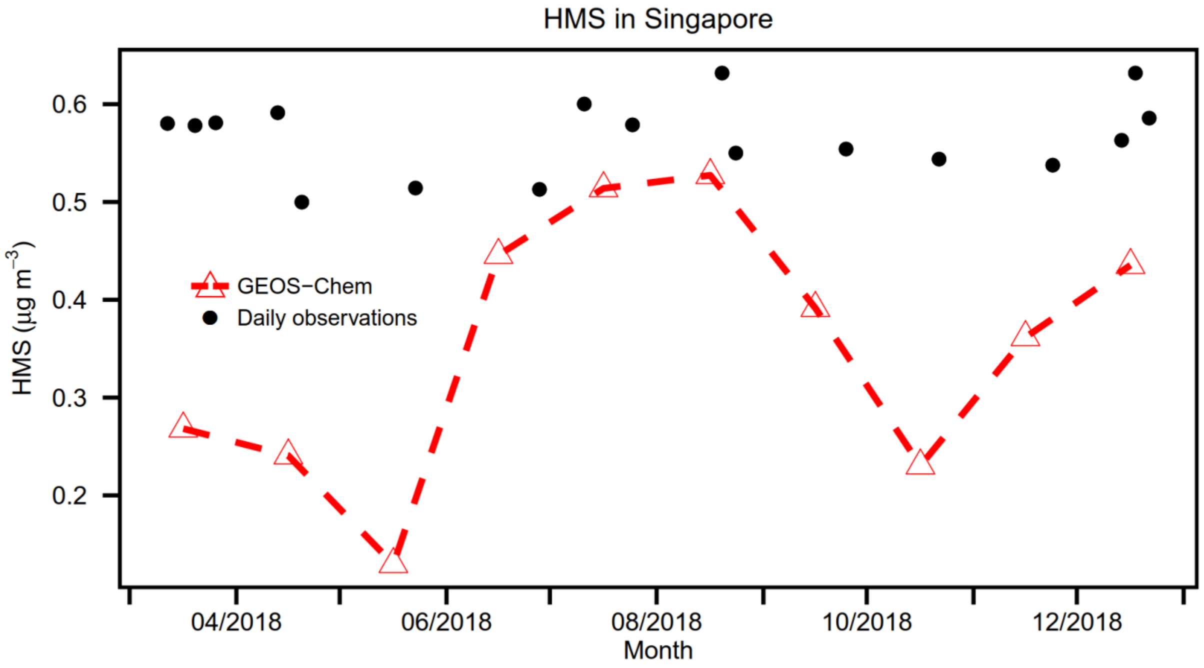

Figure S7: Observed and simulated HMS concentrations during March 2018 to December 2018 for Singapore. Open triangles connected by dashed lines represent the GEOS-Chem simulated monthly mean at 2°×2.5° resolution, and the dots represent 12-hour mean observations. Observations in Singapore are from ion chromatography performed with an AS12A column. Samples were stored for 9 to 18 months between collection and analysis.

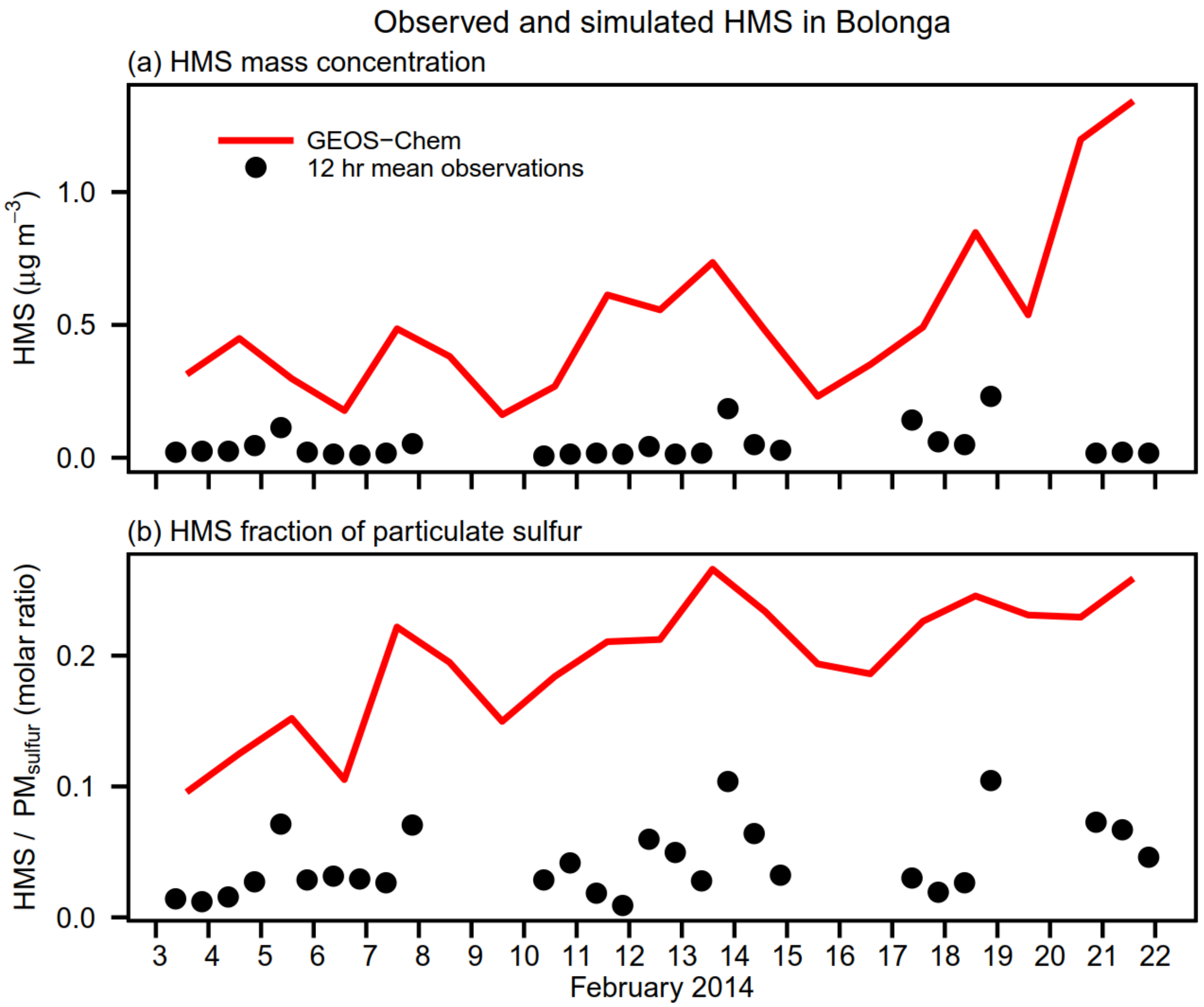

Figure S8: Observed and simulated (a) HMS concentrations and (b) molar fraction of HMS in total particulate sulfur (sulfate + HMS) during February 3-22, 2014, in Bologna, Italy. The red line indicates hourly model results from GEOS-Chem at 2°×2.5° resolution, and the black dots represent 12-hour mean observations from Bologna with ion chromatography for sulfate and NMR for HMS. Samples were stored for approximately one year between collection and analysis.

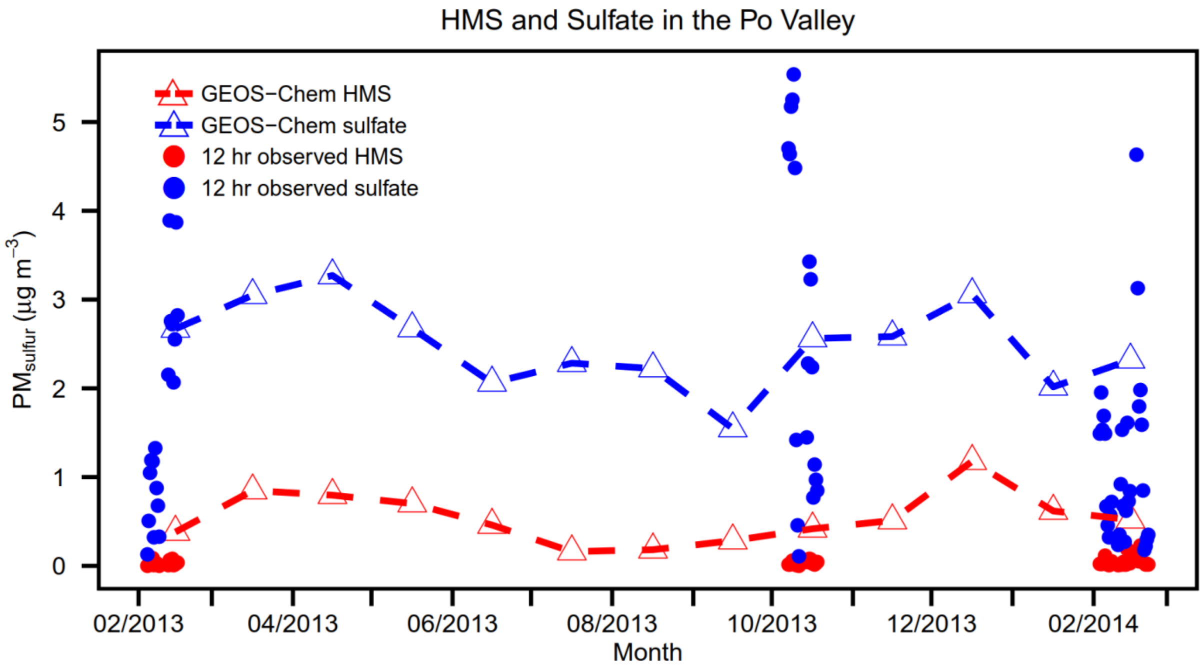

Figure S9: Observed and simulated HMS and sulfate concentrations from February 2013 to February 2014 for Bologna, Italy. The open triangles connected by dashed lines represent GEOS-Chem simulated monthly means at 2°×2.5° resolution. Dots represent 12-hour mean observations, with red corresponding to NMR measurements of HMS and blue to ion chromatography (IC) measurements of sulfate. The IC system relied on an AS11 column, which cannot efficiently separate HMS and sulfate. Samples were stored for 5 to 12 months between collection and analysis.

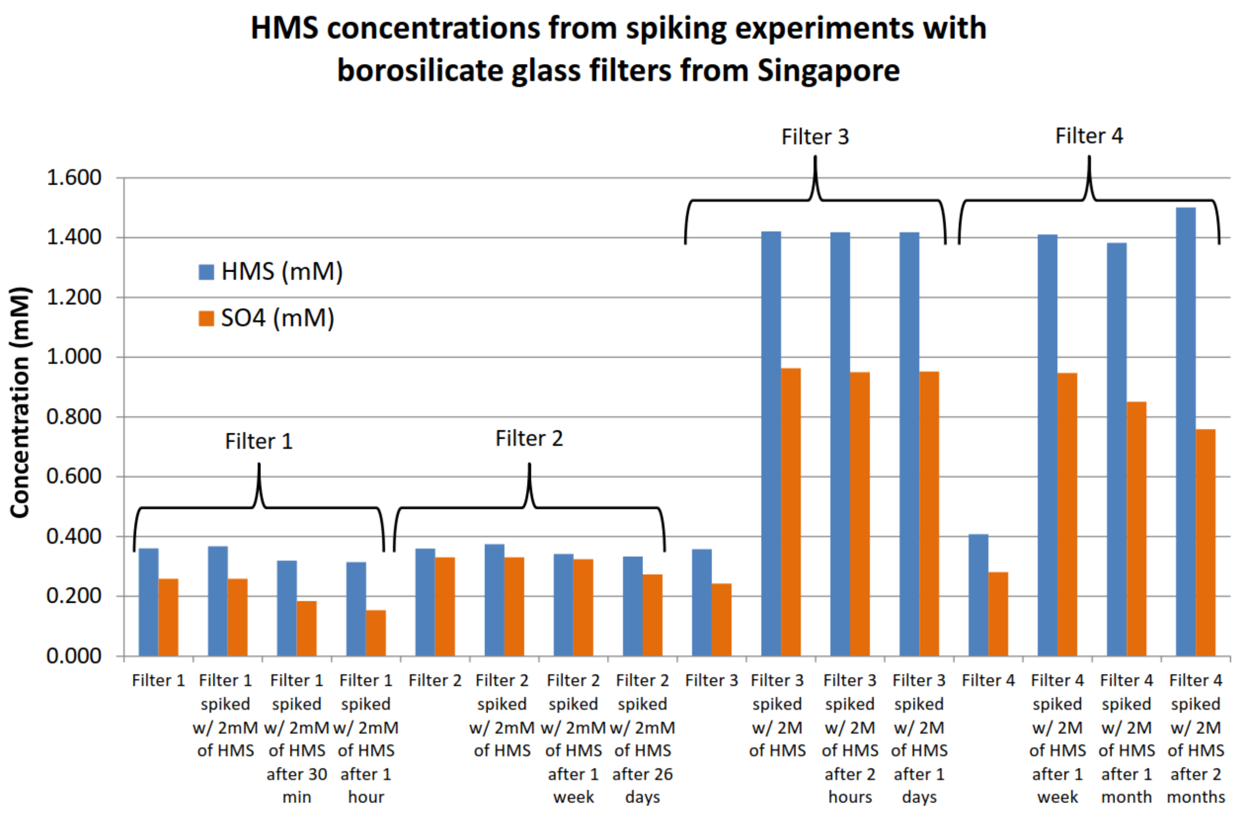

<u>Figure S10:</u> Example results for HMS spiking experiments with borosilicate glass fiber filters from Singapore. Blue bars show HMS concentrations and orange show sulfate concentrations in mM as measured by IC. Filters were spiked with solutions of various concentrations of HMS and measured immediately after spiking or after being stored between 30-minutes and 2-months. Each set of vertical bars represents the measurement of one quarter of a filter.

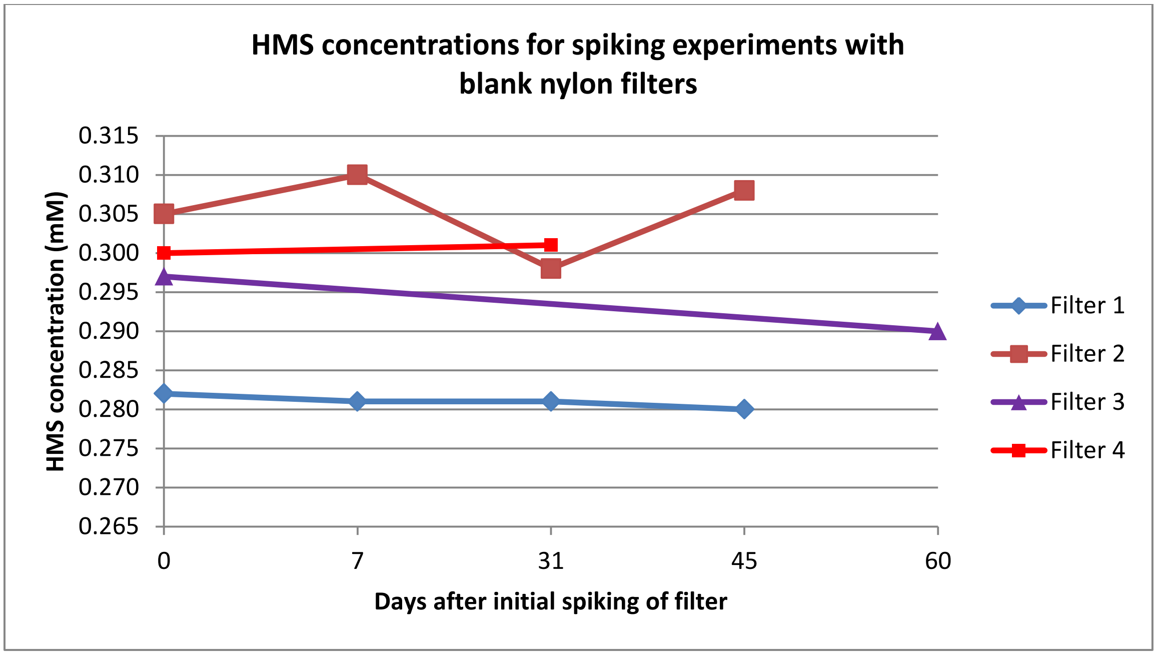

<u>Figure S11:</u> Example results for HMS spiking experiments with blank nylon filters. HMS concentrations are in mM as measured by IC. Each point represents the measured HMS on one quarter of a filter. The sulfate concentrations for all measurements shown here were either 0.001 or 0.002 mM.

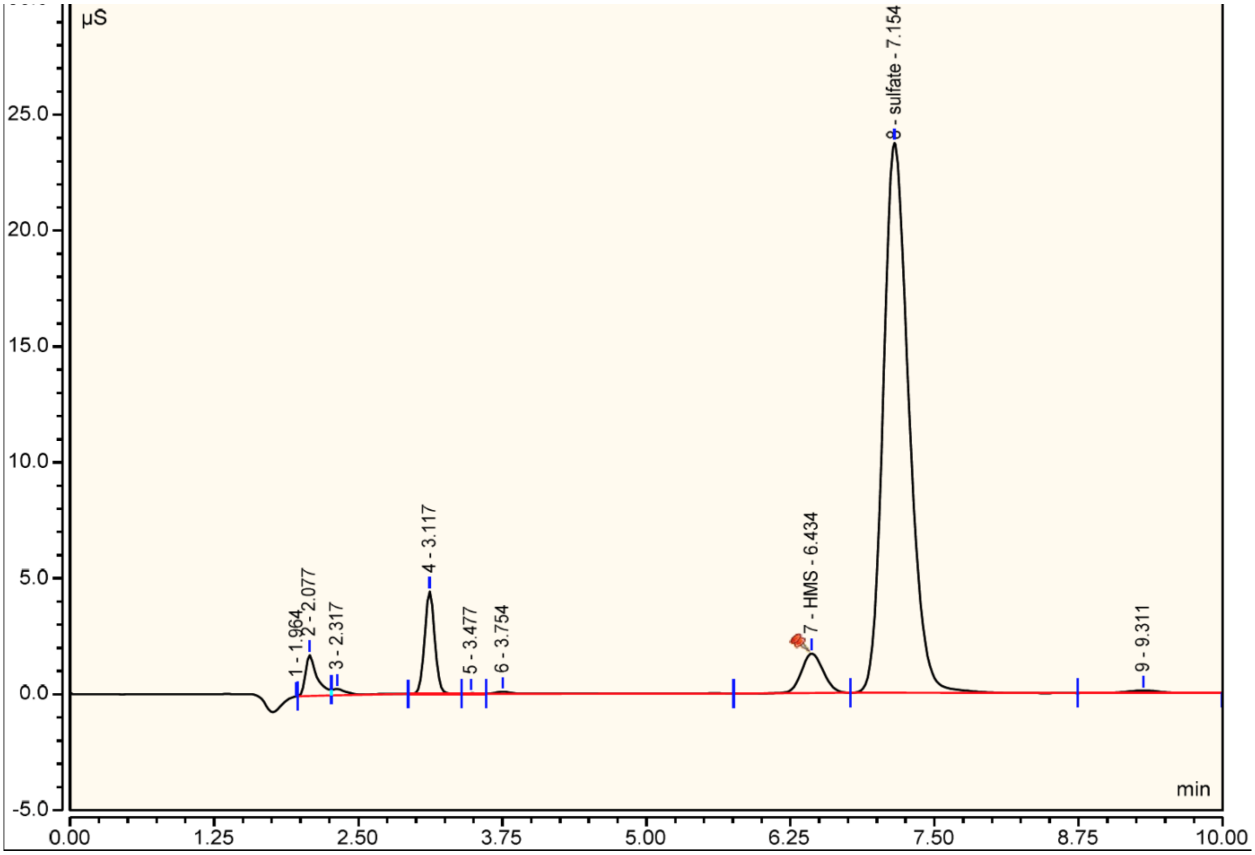

Figure S12: Example chromatogram from the decomposition experiments conducted with the borosilicate glass fiber filters from Singapore. This sample was spiked with 10mM of sulfate, and measured immediately after spiking. Measured HMS was 0.4 mM and sulfate was 2.2 mM.

References:

Cao, C., Jiang, W., Wang, B., Fang, J., Lang, J., Tian, G., et al. (2014). Inhalable Microorganisms in Beijing's PM2.5 and PM10 Pollutants during a Severe Smog Event. *Environmental Science & Technology*, *48*(3), 1499–1507. https://doi.org/10.1021/es4048472

Moch, J. M., Dovrou, E., Mickley, L. J., Keutsch, F. N., Cheng, Y., Jacob, D. J., et al. (2018). Contribution of Hydroxymethane Sulfonate to Ambient Particulate Matter: A Potential Explanation for High Particulate Sulfur During Severe Winter Haze in Beijing. *Geophysical Research Letters*, *45*(21), 11,969-11,979. https://doi.org/10.1029/2018GL079309
